# Public knowledge, attitudes and practices toward diabetes mellitus: A cross-sectional study from Jordan

**DOI:** 10.1371/journal.pone.0214479

**Published:** 2019-03-29

**Authors:** Mervat Alsous, Mariam Abdel Jalil, Mohanad Odeh, Rasha Al Kurdi, Murhaf Alnan

**Affiliations:** 1 Faculty of Pharmacy, Department of Pharmacy Practice, Yarmouk University, Irbid, Jordan; 2 Faculty of Pharmacy, Department of Biopharmaceutics and Clinical Pharmacy, The University of Jordan, Amman, Jordan; 3 Faculty of Pharmaceutical Sciences, Department of Clinical Pharmacy and Pharmacy Practice, Hashemite University, Zarqa, Jordan; 4 Faculty of Pharmacy, Department of Clinical Pharmacy and Therapeutics, Applied Science Private University, Amman, Jordan; West Virginia University, UNITED STATES

## Abstract

**Aims:**

To assess the knowledge and practices toward diabetes in the Jordanian community.

**Methods:**

This study was conducted as a public based cross-sectional study in different cities in Jordan. A previously published validated questionnaire about knowledge, attitudes, and practices (KAP) toward diabetes mellitus (DM) was translated from the Arabic version and used in this study with very minor modification to be suitable for this study of the Jordanian population.

**Results:**

A total of 1,702 participants were recruited in the present study. About half of the participants (53.3%) had good knowledge scores. The respondents’ knowledge scores were significantly correlated with attitudes (*p* < 0.001). The education level (university or higher) and education related to a field were predictors for good knowledge and positive attitudes. About 46.3% of participants had positive attitudes toward the disease. As for practices, 37.7% of participants did not engage in regular exercise while more than half of the study subjects had never checked their blood glucose level on an annual basis. The factors influencing the practice of checking blood glucose level have been investigated.

**Conclusion:**

This study has highlighted the need for more educational interventions to address negative attitudes and promote healthy lifestyle practices and regular health checks especially in certain subgroups of patients, such as those not having a degree related to the medical field and not having a first-degree relative with DM.

## 1. Introduction

Diabetes mellitus (DM) is one of the most common non-communicable illnesses worldwide [1]. The world prevalence of DM among adults is increasing and is estimated reach 7.7% by 2030 [2]. The overall prevalence of DM among adults in Jordan was 17.1% in 2008 [3]. DM is associated with many classical symptoms including increased thirst and hunger in addition to frequent urination causing serious long-term macro and micro vascular complications [4]. Moreover, untreated complications may lead to death [5].

Complications associated with DM can be reduced by the early diagnosis of the disease and proper treatment [4]. An optimal glycemic control can be achieved by regular exercise, eating a healthy diet, weight loss, and adherence to a prescribed drug therapy [4].

Consequently, knowledge about the disease and its complications plays an integral role in the management of diabetes. Patients with proper knowledge about diabetes and its complications pursue a suitable treatment and health care [6].

The use of a knowledge, attitudes, and practices (KAP) based survey is considered a good resource model to access the qualitative and quantitative information held by the individual [7]. A KAP questionnaire is used to establish the baseline value for use in future assessments and to help assess the effectiveness of health education interventions [7]. Several studies have used a KAP based questionnaire to assess the knowledge, attitudes, and practices of individuals toward the DM disease [8–13] and supported the need for better awareness of how to control the risk factors and prevent diabetes [8]. There is strong evidence that educated diabetic patients with good knowledge and health literacy achieve better disease control and management [14, 15] involving non-pharmacological treatment and pharmacological drug therapy.

There are no previous research studies conducted in Jordan to assess the KAP of the general public related to diabetes and its complications. Assessing the KAP related to diabetes mellitus in the general public would be helpful to provide a better insight to address poor knowledge about the disease and the development of potential preventive strategies specific to Jordan. Therefore, this study was commenced to assess the KAP toward diabetes among a sample of the general public in Jordan.

## 2. Methods

### 2.1 Study design

2.2 A cross-sectional study was carried out in different geographical locations of Jordan.

### 2.2 Ethical approval and recruitment procedure

Ethical approval for this study was obtained from the Ethical Review Committee of the Faculty of Medicine, Hashemite University, Zarqa, Jordan 1/4/2018/2019. About 1,702 people from the Jordanian public participated in the study. The questionnaire was distributed to the public by the authors and 2 research assistants. The target sample size was 1,000. The study was conducted in 11 different cities in Jordan between April 2018 and August 2018. Members from the public older than 18 years who are able to read and write the Arabic language were eligible to participate in the study. The eligibility for participation was assessed through the interview of the research assistant with the participant to identify individuals who may have problems understanding purpose of the study and consent-related issues. The questionnaire was uploaded on to google forms, and portable (pc)/ iPad/ tablet were used to fill the questionnaire data. Recruitment of participants was conducted in public places (i.e. hypermarkets, Malls and Gyms) in different cities in Jordan. The questionnaire was prefaced by a page explaining the nature and purpose of the study and a consent statement, participants were given the freedom to read it, and consent to take part was considered taken if the participant filled out the questionnaire and signed the consent form electronically. This procedure was approved by the IRB committee.

### 2.3 Study instrument

A previously published validated questionnaire about the knowledge, attitudes, and practices (KAP) regarding DM was obtained with permission from the authors [13]. The Arabic translated version of the validated questionnaire was used in this study with very minor modification to be suitable for researching the Jordanian population. The questionnaire was piloted utilizing a sample of 12 participants from the public; 5 of them with a history of DM and 7 participants without history of DM to ensure the face and content validity of the Arabic translated questionnaire. In addition, the Arabic version of the questionnaire was revised by two PhD holders of Clinical Pharmacy before the final version was used in the current study.

The first part of the questionnaire covered the demographic data of the respondents which included age, gender, level of education, average monthly income, and occupation. Specifically, occupation was considered related to the medical field if the participant was a physician, nurse, dentist, pharmacy staff, hospital staff, or medical laboratory staff. Students/graduates from a medical college, pharmacy, nursing, laboratory sciences, and dentistry were considered as having degrees related to the medical field.

Knowledge about diabetes was measured using eight foremost questions related to risk factors, diagnosis, prevention, and complications of diabetes mellitus. Answers were provided with three different categorical responses: “Yes”, “No,” and “Don’t know.” One point was offered for each correct response and the total score was calculated out of 26. Score ranges of 0–13, 14–18, and 19–26 were categorized as having poor, moderate, and good knowledge, respectively.

The attitude of the public toward diabetes was assessed using seven questions related to adherence to the treatment of DM. Responses of participants were assessed with categorical responses: “Yes”, “No,” and “Don’t know.” Participants who got ≥4 marks out of seven were categorized as having positive attitudes.

Practices toward DM were assessed using four questions. Responses to the individual questions were classified into positive and negative practices. Examples of these questions were "Would you consider treatment if you or one of your family members is found to have diabetes?" and "Do you check your blood sugar regularly (at least annually)?" Answers were provided with three different categorical responses: “Yes”, “No, and “Don’t know.” If respondents answered yes, then they were classified as having a positive practice, and if the answer was no, they were classified as having a negative practice.

Example of the modified questions suitable for the Jordanian public is about the type of herbals used by people, such as cinnamon, fenugreek, ginger, as these are known to be used among the Jordanian public while in the original questionnaire, Thebu leaves (Costus speciosus) or Karivila (Momordica dioica) were used [13] and these are not well known in Jordan.

### 2.4 Statistical analysis

Data were analyzed using the statistical package for science (SPSS), version 24. Participants’ demographic data including gender, age, level of education, and income were reported using descriptive statistics. Univariate analysis was done using the t-test for continuous variables and chi-square for categorical variables. Binary logistic regression was done using Backward LR, and *p*-value was considered significant if its value was < 0.05.

The questionnaire’s internal consistency was assessed by measuring the Cronbach alpha coefficient for questionnaire scales. The test results were participants’ knowledge about DM, 0.743; participants’ attitude toward DM, 0.746; and participants’ practices toward DM, 0.617.

## 3. Results

### 3.1 Demographic characteristics

Out of 1,702 of total participants, 69.73% were females and the mean age was 32.15 ± 12.75 years. About 41% of participants completed studies related to the medical field. About half of the participants have first-degree relatives with DM. Data are presented in Table 1.

**Table 1:** Respondent’s demographic characteristics (N = 1702).

	Number	%
**Age**		
• <40 years	1296	76.1
• ≥40 years	406	23.8
**Gender**		
• Male	522	30.7
• Female	1180	69.3
**Level of Education**		
• Up to secondary school	223	13.1
• College	131	7.7
• University	1169	68.7
• Master	130	7.6
• PhD	49	2.9
**Education related to medical field**		
• Yes	698	59.0
• No	1004	41.0
**Have first degree relative with Diabetes Mellitus**		
• Yes	888	52.2
• No	725	42.6
• Do not know	89	5.2
**Income**		
• <500 JD	884	51.9
• 500–1000 JD	448	28.5
• >1000JD	334	19.6
**Marital status**		
• Single	905	53.2
• Married	774	45.5
• Divorced	23	1.4
**Nationality**		
• Jordanian	1372	80.6
• Not Jordanian	330	19.4
**Place of living**		
• Amman	1090	64.0
• Other provinces in Jordan	420	24.7
• Outside Jordan	192	11.3

### 3.2 Knowledge assessment

Knowledge was measured using eight questions related to disease diagnosis, risk factors prevention, and complications. The mean knowledge score for participants was 17.9 ± 4.14.

**Fig 1:**
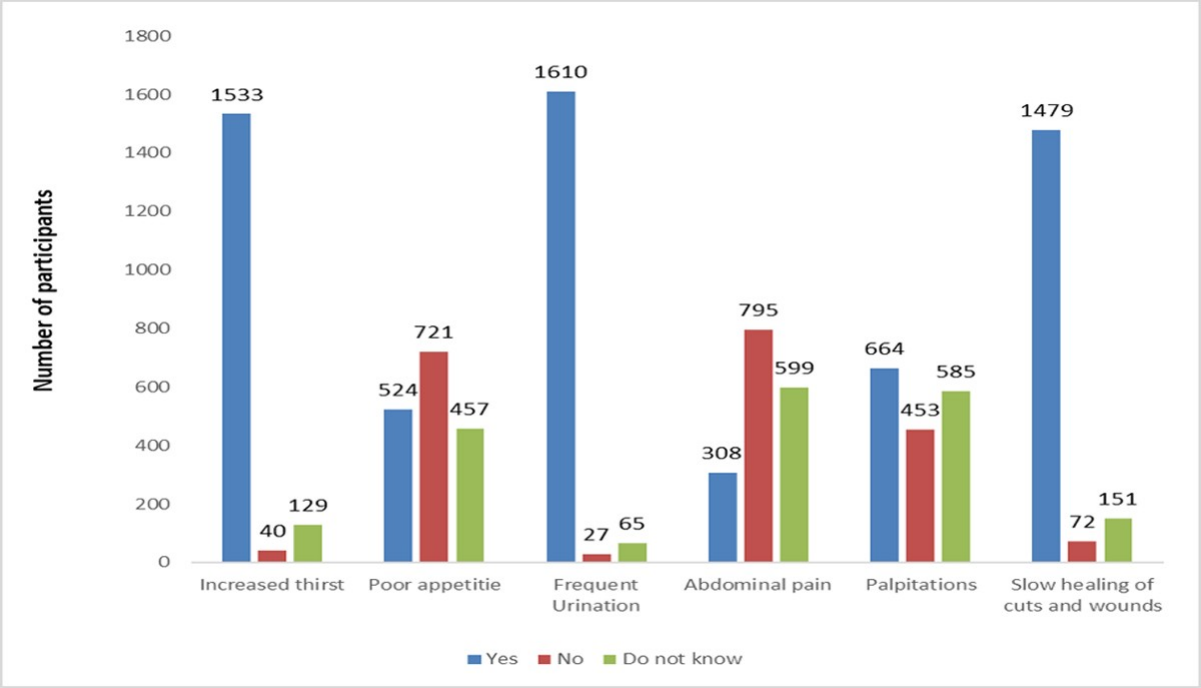
Participants knowledge about classical symptoms of Diabetes Mellitus (N = 1702).

About 53.5% of participants had good knowledge scores. Only 13.1% of participants scored less than 14 which corresponds to a poor level of knowledge. Most of the participants (81.3%) were aware that the dysfunction of the pancreas leads to DM. Among symptoms of the disease, the most reported one was frequent urination (94.6%) followed by increased thirst (90.1%) and slow healing of wounds (86.9%). See Fig 1.

When participants were asked if organs may be affected by DM, 87.8% of them agreed that they may, and the kidney was the most frequently reported organ affected by the disease. However, the majority of participants (20.15%) were unaware that DM can affect the heart (Table 2).

**Table 2:** Participants’ knowledge on DM complications (N = 1702).

	Yes (%)	No (%)	Do not know (%)
Stroke	46.48	18.5	35.02
Heart Attack	45.24	20.15	34.61
Hepatitis	32.96	28.5	38.54
Kidney Failure	68.98	8.4	22.62
Arthritis	40.83	24.56	34.61

The association of socioeconomic factors with a good knowledge score (≥ 19) was assessed using the Chi-square analysis, and there was no significant association between the factors of gender or marital status and the knowledge score (*p*-value > 0.05) while the factors of a high level of education, having first-degree relatives with DM, income higher than 800 JD, and an education related to the medical field were significantly associated with a good knowledge score (*p*-value < 0.05).

Significant factors were subjected to the binary logistic regression analysis and results showed that only an education related to the medical field, a university level of education or higher, and having first-degree relatives with DM were predictors of a good knowledge score (Table 3).

**Table 3:** Predictors of participants’ good knowledge about DM using binary logistic regression^8^.

Independent variable	B	SE	Odds ratio	95% CI	*p*-value
Income higher than 800 JD	0.22	0.12	1.25	0.99–1.58	0.061
Education related to medical field	1.75	0.12	5.75	4.56–7.25	<0.001*
University level of education or higher	0.28	0.14	1.33	1.01–1.74	0.040*
Having first degree relative with Diabetes Mellitus	0.55	0.11	1.73	1.40–2.14	<0.001*

*p-value < 0.05.

^**8**^ Poor and moderate knowledge score coded 0, good knowledge score coded 1.

B, regression coefficient; SE, standard error associated with the coefficient B; CI, confidence interval.

### 3.3 Attitude assessment

The attitudes of the public toward DM were assessed using seven questions and those who scored < 3 points were considered as having negative attitudes while those who scored ≥ 4 points were considered as having positive points.

**Fig 2:**
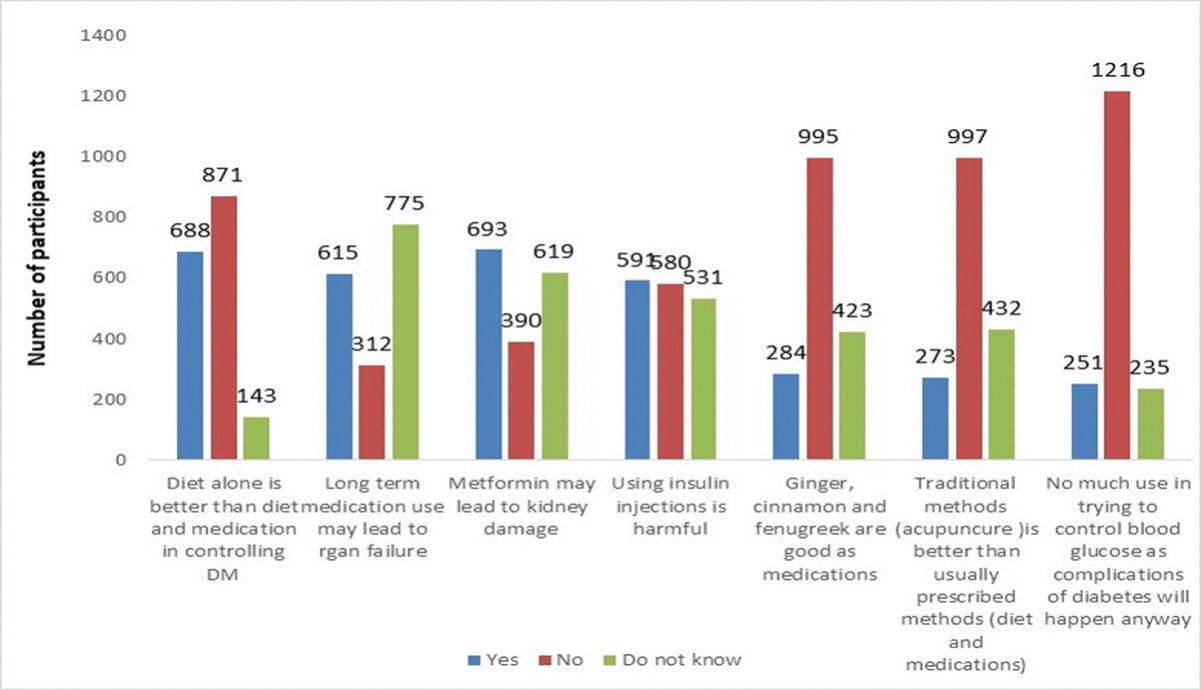
Participants responses toward attitude questions (N = 1702).

About 46.3% of participants had positive attitudes toward the disease. One third of the participants (34.7%) believed that the use of insulin is harmful to the body. Around 40% of participants believed that the long-term use of anti-diabetic agents cause organ failure and that glucose can be controlled by having the right diet better than using medications. Almost 16% believed that using complementary therapy, herbal remedies (cinnamon, fenugreek, ginger) and alternative medicine, such as acupuncture, were better in controlling blood glucose level than using medications and diet (Fig 2).

The respondent’s knowledge score was significantly correlated with the attitude score (Pearson correlation, 0.436, *p*-value < 0.001).

A univariate analysis was used to find factors associated with positive attitudes, and results showed that there was no significant association with the factors of age, gender, and marital status. However, the factors of education level (university or higher) and education related to medical field were significantly associated with positive attitudes (*p*-value < 0.05). Following binary logistic regression analysis, both variables were predictors of positive attitudes among the public (Table 4).

**Table 4:** Predictors of participants’ positive attitudes toward DM using binary logistic regression^8^.

Independent variable	B	SE	Odds ratio	95% CI	*p*-value
Education related to medical field	1.31	0.11	3.71	2.99–4.59	<0.001*
University level of education or higher	0.49	0.14	1.64	1.25–2.15	<0.001*

*p-value < 0.05.

^8^ negative attitudes coded 0, positive coded 1.

B, regression coefficient; SE, standard error associated with the coefficient B; CI, confidence interval.

### 3.4 Practice assessment

**Fig 3:**
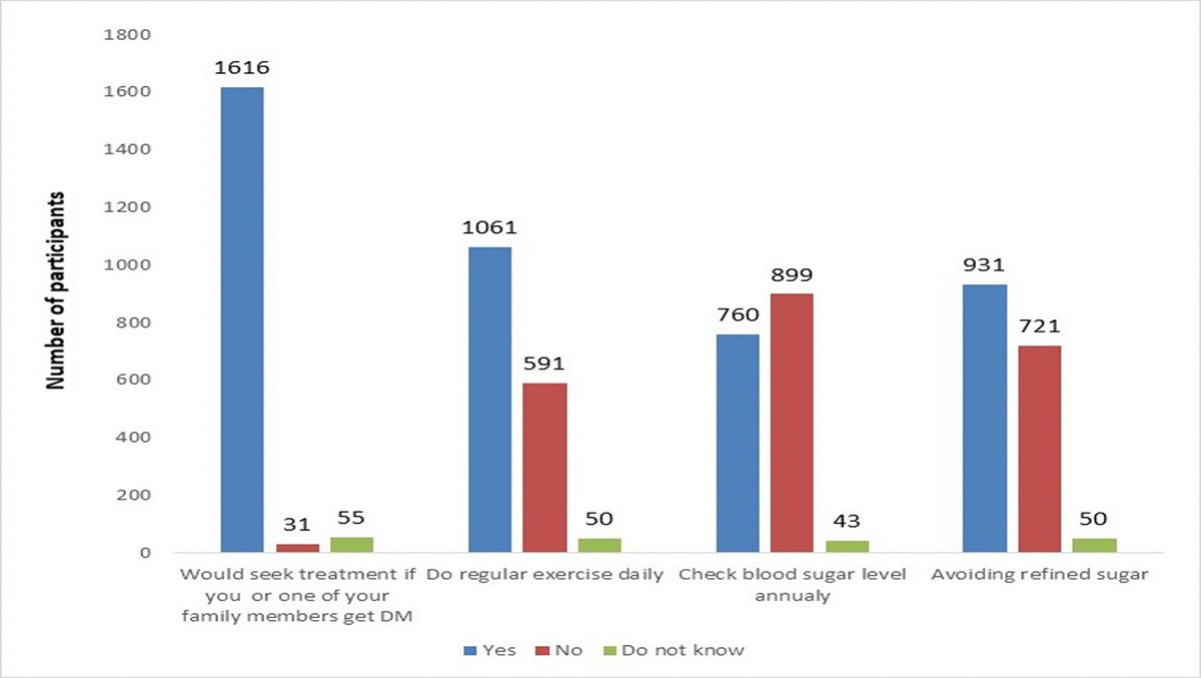
Practices assessment toward Diabetes among the public in Jordan (N = 1702).

Practices toward DM were evaluated using four questions regarding the participants intention to seek treatment, do preventive measures such as exercising or eating healthy food, and do annual screening for the disease. Most of participants (94.9%) stated that they would seek treatment if they or one of their family members get DM. However, a lesser proportion of participants (62.3%) do regular exercise. More than half of the study subjects had never checked their blood glucose level on an annual basis. About 45.3% of participants use refined sugar (Fig 3). The mean score of participants’ knowledge was higher among those with positive practices in regards to doing exercise regularly compared to those who do not do these practices (*p*-value < 0.001, Table 5). After analyzing the association of factors affecting participants’ practice of screening for DM annually, those with an education related to the medical field, older than 40 years, and having first-degree relatives with DM had regular blood sugar measurements (Table 6).

**Table 5:** Respondents’ Knowledge score among those with positive and negative practices toward DM (N = 1702).

	Mean Knowledge score ± SD	*p*-value
Would seek treatment if himself or a family member get Diabetes Mellitus		
• Yes	18.19+3.94	<0.001*
• No	14.35+5.78	
Do regular exercise		
• Yes	18.54+3.61	<0.001*
• No	17.09+4.75	
Check blood sugar annually		
• Yes	19.06+3.50	<0.001*
• No	17.14+4.75	
Avoiding refined sugar		
• Yes	18.63+3.81	<0.001*
• No	17.22+4.38	

**Table 6:** Factors affecting participants’ practice to check blood regularly using binary logistic regression^8^.

Independent variable	B	SE	Odds ratio	95% CI	*p*-value
Education related to medical field	0.69	0.11	1.99	1.61–2.46	<0.001*
Having first degree relative with DM	0.70	0.10	2.02	1.66–2.47	<0.001*
Age more than 40 years	0.79	0.13	2.21	1.73–2.82	<0.001*

*p-value < 0.05.

^8^ Poor and Do not check blood glucose level for DM annually coded 0, Check blood glucose level for DM annually coded 1.

B, regression coefficient; SE, standard error associated with the coefficient B; CI, confidence interval.

## 4. Discussion

The global status report on non-communicable diseases published in 2014 by the World Health Organization (WHO) reported that in 2012 diabetes was responsible for 1.5 million deaths and 89 million disability-adjusted life-years [16]. Globally, the diabetes prevalence in 2014 was estimated to be 9% (based on a fasting glucose level greater than 126 mg/dl or receiving medications for the purpose of lowering blood glucose) [16]. In the Eastern Mediterranean region, which includes Jordan, the prevalence of diabetes is even higher than the global rate and reaches to 14% [16]. This high prevalence, coupled with the long-term consequences of diabetes, makes it imperative for health care givers to design prevention and management plans for this disease. Such plans should be based on the baseline knowledge, attitudes, and practices of the target population.

The high percentage of educated participants (BSc or higher) in the present sample (68.7%) reflects the high level of education in the Jordanian community in general. This is evident from the 2015 SABER (Systems Approach for Better Education Results) country report that revealed that the 2010 illiteracy in Jordan was the lowest among the Arab world countries [17]. Furthermore, the transition rate to higher education varied between 79 and 85 percent of secondary school graduates between 2005 and 2009 [17]. The average score of the Jordanian population in regards to knowledge was 17.9 ± 4.14 which is classified as the moderate level; the source of this knowledge however was not assessed in the present study. This score was similar to that reported in a small study conducted in Sri Lanka which was 16.5 ± 0.51. On the other hand, several studies from developing countries reported poor knowledge of diabetes among the general public [8, 18–20].

An accurate comparison of the knowledge results in this study to others might be difficult due to differences in the target population (most researchers investigate knowledge in diabetic patients) [18,21] or differences in the scoring systems or cut-off points that discriminate between different levels of knowledge [22].

The present study showed a significant association between the level of education and knowledge which was similar to many other studies [11,13,19,23].

Having first degree relatives with diabetes mellitus can have a strong impact on the knowledge regarding diabetes as pointed out by our study and the study of Al-Maskari et al [18]. This is due to the fact that people with a family history of a chronic disease, such as hypertension or diabetes, can develop a delicate sense of vulnerability that could increase their level of awareness about the disease [24]. This was also reflected in their practices as they were more likely to have annual blood glucose tests.

About half of the respondents had a positive attitude (46.3%). The participants’ knowledge was significantly correlated with the attitude meaning that increased knowledge is related to positive attitudes. Alarmingly, a significant proportion of the study participants believed that taking insulin was harmful (34.7%) and oral anti-diabetic agents cause renal damage (40.0%). These wrong beliefs too can directly affect the management of diabetes in Jordanian society. Therefore, it is essential to direct more interventions to improve the knowledge and develop an educational model to reinforce the attitude of the general public.

A significant proportion of the respondents (16%) reported that the use of complementary and alternative medicine (CAM) may achieve better glycemic control as compared to medications. Countries in the Arab region, such as Saudi Arabia and Bahrain, have reported a high usage of CAM by diabetic patients at 30.1% and 63.0%, respectively [25,26]. The use of CAM in the management of diabetes without any medical supervision can be a dangerous practice. We do not know to what degree patients disclose their use of CAM, but future educative efforts should increase the awareness about the positive and negative effects of the use of CAM in the management of many chronic diseases including diabetes, in addition to encouraging patients to disclose their CAM use with their health care provider.

Participants reporting positive practices such as exercise had a greater level of knowledge in this study, but it is important to highlight that positive knowledge might not always translate to positive practices. For instance, a study conducted by Al-Tamimi and Petersen revealed that although mothers of school children were aware of the harmful effects of candy on teeth, the consumption of sugary food was high [27]. Therefore, future efforts should focus on increasing the awareness about the disease and reinforcing positive practices.

In the current study, more than half of the study subjects had never checked their blood glucose level on an annual basis. Participants that were older than 40 and/or had an education related to the medical field were significantly associated with the practice of screening for DM annually. As people get older, they might be more aware or concerned about their health which could lead to doing more tests, in general, including blood sugar tests. It is important to note that doing such health checks can be expensive for certain people, and thus they could consider it as an unnecessary luxury.

## 5. Limitations

A large proportion of the study population had an education related to medical field, although the Jordanian population has the highest per-population rates of health care professionals in the Middle East [28], this does not exclude the possibility bias with more health aware people being included in the study. Therefore, the results may not be actually representative of general the public. Furthermore, we did not examine the sources of health information. Knowledge of the sources of knowledge would have been useful in identifying the most appropriate intervention for health promotion among the general public in Jordan.

## 6. Conclusions

In conclusion, despite limitations, the present study comprehensively assessed the general public’s knowledge, attitude, and practices toward DM in Jordan. The sample size used in this study gives it the strength to report reliable and representative results. To our knowledge this is the first study to explore these issues in Jordan. This study has highlighted the need for more educational interventions to promote healthy lifestyle practices and regular health checks especially in certain subgroups of patients, such as those not having a degree related to the medical field and not having first degree relatives with DM.

## Supporting information

S1 File Questionnaire. (DOCX)

S2 File Data. (SAV)

S3 File English translated questionnaire. (DOCX)
