# Supplementary material for: Public knowledge, attitudes and practices toward diabetes mellitus: A cross-sectional study from Jordan
Source: PLoS One. 2019 Mar 29;14(3):e0214479. doi: 10.1371/journal.pone.0214479 (PMC6440628; doi:10.1371/journal.pone.0214479)
Supplement: S1 File — (DOCX) [file pone.0214479.s001.docx]

**نموذج موافقة للمشاركة في دراسة لمرض السكري**

**تهدف هذه الدراسة إلى تقييم معلومات المشتركين حول مرض السكري ، معلومات المشتركين الشخصية ستكون سرية ولن تنشر . إذا كنت توافق على الإشتراك في الدراسة يرجى تعبأة الاستبيان المرفق.**

**⸋ موافق**

**⸋ غير موافق**

**الجزء الأول - المعلومات الديموغرافية**

| اسم المشترك (يمكن استخدام اسم مختصر أورمز بدل الاسم الصريح) |
| --- |
| العمر: |
| الجنسية: |
| طبيعة العمل: |
| هل تعمل في المجال الطبي(مثلا تعمل في مستشفى-مستوصف صحي-صيدلية-مختبر طبي)  : ⸋ نعم ⸋ لا  ما هو : |
| مكان السكن :   - **الوسط:** ⸋عمان ⸋ البلقاء /السلط ⸋الزرقاء ⸋ مادبا - **الشمال:** ⸋اربد ⸋ عجلـــــــــون ⸋ جرش ⸋ المفرق - **الجنوب:** ⸋معان ⸋الكـــــــــرك ⸋ الطفيلة ⸋العقبة - غير ذلك.................................................. |
| الجنس:   - ذكر - أنثى |
| الحالة الاجتماعية:   - أعزب - متزوج - مطلق |
| مستوى التعليم:   - غير مُتعلَم - ابتدائي - إعدادي - ثانوي - كلية مجتمع (معهد ) - جامعة - ماجستير - دكتوراة   *في حال كان التعليم كلية مجتمع / جامعة / ماجستير / دكتوراه هل طبيعة التخصص متعلقة بالمجال الطبي (مثلا كلية الطب-طب الاسنان-صيدلة- التمريض-العلوم المخبرية)  ( ⸋ نعم ⸋لا ) |
| مُعدَل الدخل الشهري بالدينار الأردني:   - أقل من 200 - 200-500 - 501-800 - 801-1000 - أكثر من 1000 |
| مريض السكري قريب من الدرجة الأولى (الأب، الأم، الابن أوالبنت):   - نعم - لا - لا أعرف |

الجزء الثاني - المعرفة

| السؤال |  | | | |
| --- | --- | --- | --- | --- |
| ما هو مرض السكري؟ |  |  |  |  |
| 1. ماذا يحدث لمستوى السكر في الدم عند مريض السكري؟ | **لا يتغير** | **يرتفع** | **ينخفض** | **لا أعرف** |
| 1. أي من الأعضاء التالية إذا حدث فيها خلل فإنَ ذلك يؤدي للإصابة بمرض السكري   أرجو التحديد إذا كنت تعرف: |  | **نعم** | **لا** | **لا أعرف** |
| - **الرئة** |  |  |  |  |
| - **الكلى** |  |  |  |  |
| - **البنكرياس** |  |  |  |  |
| - **الكبد** |  |  |  |  |
| - **الدماغ** |  |  |  |  |
| 1. هل من الممكن أن يتعافي مريض السكري من المرض بشكل نهائي؟ |  | **نعم** | **لا** | **لا أعرف** |
| 1. أي من العوامل الآتية تزيد من خطر الاصابة بمرض السكري؟ |  | | | |
| - التاريخ العائلي لمرض السكري |  | **نعم** | **لا** | **لا أعرف** |
| - زيادة الوزن او البدانة |  | **نعم** | **لا** | **لا أعرف** |
| - تناول السكريات بكثرة |  | **نعم** | **لا** | **لا أعرف** |
| - الجلوس لساعات طويلة في العمل أو المنزل مع عدم ممارسة الرياضة بشكل كافي |  | **نعم** | **لا** | **لا أعرف** |
| - الضغط النفسي |  | **نعم** | **لا** | **لا أعرف** |
| أعراض مرض السكري |  | | | |
| 1. أي من الأعراض التالية هي أعراض معتادة تحدث عند مريض السكري؟ |  |  |  |  |
| - زيادة العطش |  | **نعم** | **لا** | **لا أعرف** |
| - فُقدان الشهية |  | **نعم** | **لا** | **لا أعرف** |
| - التبول المتكرر |  | **نعم** | **لا** | **لا أعرف** |
| - وجع البطن |  | **نعم** | **لا** | **لا أعرف** |
| - خفقان القلب (بسبب ارتفاع السكر) |  | **نعم** | **لا** | **لا أعرف** |
| - بُطء الشفاء من الجروح |  | **نعم** | **لا** | **لا أعرف** |
| علاج مرض السكري |  | | | |
| 1. **أي من العلاجات التالية فعالة في السيطرة على نسبة السكر في الدم؟** |  |  |  |  |
| - **حقن الانسولين** |  | **نعم** | **لا** | **لا أعرف** |
| - **الأدوية الفموية** |  | **نعم** | **لا** | **لا أعرف** |
| - **ممارسة الرياضة بشكل مستمر** |  | **نعم** | **لا** | **لا أعرف** |
| - تجنَب الأطعمة السكرية |  | **نعم** | **لا** | **لا أعرف** |
| - تناول الأعشاب , الزنجبيل والقرفة بشكل منتظم |  | **نعم** | **لا** | **لا أعرف** |
| 1. **هل تعتقد أن مرض السكري يُمكن أن يُؤثًر على الأعضاء الأخرى؟**   **إذا كانت الإجابة بـ "نعم" ، أي من المضاعفات التالية يمكن أن تحدث بسبب مرض السكري؟** |  | **نعم** | **لا** | **لا أعرف** |
| - السكتة الدماغية |  | **نعم** | **لا** | **لا أعرف** |
| - نوبة قلبية |  | **نعم** | **لا** | **لا أعرف** |
| - **التهاب الكبد** |  | **نعم** | **لا** | **لا أعرف** |
| - **الفشل الكلوي** |  | **نعم** | **لا** | **لا أعرف** |
| - التهاب المفاصل |  | **نعم** | **لا** | **لا أعرف** |
| 1. **ما هي أفضل طريقة لتشخيص مرض السكري ؟** |  | | | |
| - **قياس نسبة السكر في البول تعتبر هي أفضل طريقة لتشخيص مرض السكري** |  | **نعم** | **لا** | **لا أعرف** |
| - **قياس نسبة السكر في الدم بعد الصيام تعتبر هي أفضل طريقة لتشخيص مرض السكري** |  | **نعم** | **لا** | **لا أعرف** |

**الجزء الثالث – السلوك**

| 1. "هل تعتقد أنَ السيطرة على نسبة السكر مع اتبَاع نظام غذائي وحده هي أفضل من السيطرة على نسبة السكر باستخدام النظام الغذائي والأدوية"؟ | **نعم** | **لا** | **لا أعرف** |
| --- | --- | --- | --- |
| 1. "هل استخدام دواء الميتفورمين على المدى الطويل يُسبَب تلف للكلى"؟ | **نعم** | **لا** | **لا أعرف** |
| 1. "هل يُسبَب تعاطي أدوية السكري على المدى الطويل فشل في أعضاء الجسم"؟ | **نعم** | **لا** | **لا أعرف** |
| 1. "هل يسبب الأنسولين آثارا ضارة على الجسم"؟ | **نعم** | **لا** | **لا أعرف** |
| 1. "هل تعتقد أنَ استخدام الزنجبيل, القرفة, و الحلبة هو أفضل لعلاج مرض السكري من الأدوية التي يصفها الأطباء"؟ | **نعم** | **لا** | **لا أعرف** |
| 1. "هل تعتقد أنَ العلاجات البديلة (الوخز بالإبر، والعلاجات بتقويم العمود الفقري، واليوغا، والتنويم المغناطيسي، تمارين الاسترخاء أو العلاجات العشبية هي أفضل من الطرق الموصوفة عادة (مراقبة النظام الغذائي والأدوية)"؟ | **نعم** | **لا** | **لا أعرف** |
| 1. "هل تعتقد أنه ليس هناك جدوى في محاولة الحصول على تحكَم في نسبة السكر في الدم بشكل جيد، لأنَ مضاعفات مرض السكري سوف تحدث على أي حال"؟ | **نعم** | **لا** | **لا أعرف** |

**الجزء الرابع – الممارسة**

| هل تُفكَر في العلاج إذا وُجد أنَك أنت أو أحد أفراد عائلتك مُصاب بالسكري؟ | **نعم** | **لا** | **لا أعرف** |
| --- | --- | --- | --- |
| هل تقوم بممارسة 30-60 دقيقة من النشاط البدني يومياً؟ مثلاً المشي السريع، والأنشطة المنزلية، وصعود الدرج | **نعم** | **لا** | **لا أعرف** |
| هل تفحص نسبة السكر في الدم بشكل منتظم (سنوياً على الأقل)؟ | **نعم** | **لا** | **لا أعرف** |
| هل تحاول تجنَب السكر المكرر/ الأطعمة السكرية؟ | **نعم** | **لا** | **لا أعرف** |

**_شكراً جزيلاً!_**
