# Supplementary material for: Public knowledge, attitudes and practices toward diabetes mellitus: A cross-sectional study from Jordan
Source: PLoS One. 2019 Mar 29;14(3):e0214479. doi: 10.1371/journal.pone.0214479 (PMC6440628; doi:10.1371/journal.pone.0214479)
Supplement: S3 File — (DOCX) [file pone.0214479.s003.docx]

**Consent form**

The aim of this study is to assess the participants' information about diabetes. The personal information of the participants (names) will be confidential and will not be published. If you agree to participate in the study please fill out the attached questionnaire.

**⸋ Agree**

**⸋ Disagree**

**Part I - Demographic Information**

| Participant Name (or initials) | | |
| --- | --- | --- |
| Age (years): | | |
| Nationality: | | |
| Occupation: | | |
| Are you working in the medical field (for example, working in a hospital - health clinic - pharmacy - medical laboratory)?   - Yes - No   What is it: | | |
| Place of residence: |  | |
| - Irbid | - Ma'an | - Tafeelah |
| - Amman | - Jerash | - Aqaba |
| - Salt | - Ajloun | - Another province in Jordan |
| - Zarqa | - Mafraq | - Outside Jordan |
| - Madaba | - Karak |  |
| Gender:   - Male - Female | | |
| Marital status:   - Single - Married - Divorced - Widowed | | |
| Highest level of education   - Unable to read and write – no formal school education - Primary school - Middle school - Secondary school - College (Diploma) - University - Master - PhD - * In case your higher educational level is College/ University /MSc /PhD degree, whether the specialization is related to the medical field (eg Faculty of Medicine, Dentistry, Pharmacy, Nursing, Laboratory Sciences) - Yes - No | | |
| Average monthly income in Jordanian Dinar:   - <200 - 200-500 - 501-800 - 801-1000 - >1000 | | |
| First degree relatives with diabetes:   - Yes - No - Don't know | | |

**Part II - Knowledge**

| **Question** |  | | | |
| --- | --- | --- | --- | --- |
| **Diabetes disease** |  |  |  |  |
| 1. **What happens to blood sugar in diabetes?** | **No change** | **Increase** | **Decrease** | **Don't know** |
| 1. **Dysfunction of which of the following organs leads to DM? If knows,** |  | **Yes** | **No** | **Don't know** |
| - **Lung** |  |  |  |  |
| - **Kidney** |  |  |  |  |
| - **Pancreas** |  |  |  |  |
| - **Liver** |  |  |  |  |
| - **Brain** |  |  |  |  |
| 1. **Is Diabetes curable with treatment**? |  | **Yes** | **No** | **Don't know** |
| 1. **Which of the followings are risk factors of DM?** |  | | | |
| - Family history of diabetes mellitus |  | **Yes** | **No** | **Don't know** |
| - Being overweight /Obesity |  | **Yes** | **No** | **Don't know** |
| - Eating too much sugar |  | **Yes** | **No** | **Don't know** |
| - Sedentary life (or not getting enough exercise) |  | **Yes** | **No** | **Don't know** |
| - Stress |  | **Yes** | **No** | **Don't know** |
| **Symptoms of DM** |  | | | |
| 1. **Which of the followings are usual symptoms seen in diabetic patient?** |  |  |  |  |
| - Increased thirst |  | **Yes** | **No** | **Don't know** |
| - Poor appetite |  | **Yes** | **No** | **Don't know** |
| - Frequent urination |  | **Yes** | **No** | **Don't know** |
| - Abdominal pain |  | **Yes** | **No** | **Don't know** |
| - Palpitation (due to high blood sugar) |  | **Yes** | **No** | **Don't know** |
| - Slow healing of cuts and wounds |  | **Yes** | **No** | **Don't know** |
| **Management of DM** |  | | | |
| 1. **Which of the following therapies are effective in controlling blood sugar?** |  |  |  |  |
| - Insulin injection |  | **Yes** | **No** | **Don't know** |
| - Oral medications |  | **Yes** | **No** | **Don't know** |
| - Regular Exercise |  | **Yes** | **No** | **Don't know** |
| - Avoiding sugary foods |  | **Yes** | **No** | **Don't know** |
| - Regular eating of (herbs, ginger and cinnamon) |  | **Yes** | **No** | **Don't know** |
| 1. **Do you think diabetes can affect other organs?** |  | **Yes** | **No** | **Don't know** |
| - Stroke |  | **Yes** | **No** | **Don't know** |
| - Heart attack |  | **Yes** | **No** | **Don't know** |
| - Hepatitis |  | **Yes** | **No** | **Don't know** |
| - Kidney failure |  | **Yes** | **No** | **Don't know** |
| - Arthritis |  | **Yes** | **No** | **Don't know** |
| 1. **What is the best way to diagnose DM?** |  | | | |
| - Measuring urine sugar is the best way to diagnose diabetes |  | **Yes** | **No** | **Don't know** |
| - Measuring blood glucose after fasting is the best way to diagnose diabetes |  | **Yes** | **No** | **Don't know** |

**Part-III Attitude**

| 1. Do you think that controlling glucose with diet alone is superior to that of controlling glucose with diet and medications? | **Yes** | **No** | **Don't know** |
| --- | --- | --- | --- |
| 1. Can long term use of metformin cause kidney damage? | **Yes** | **No** | **Don't know** |
| 1. Does long term drug use cause organ failure? | **Yes** | **No** | **Don't know** |
| 1. Does insulin cause harmful effects to the body? | **Yes** | **No** | **Don't know** |
| 1. Do you think the use of ginger, cinnamon, and fenugreek is better for treating diabetes than prescription drugs? | **Yes** | **No** | **Don't know** |
| 1. Do you think that alternative therapies (acupuncture, yoga, hypnosis, relaxation exercises or herbal remedies are better than the methods usually prescribed (diet control and medication)? | **Yes** | **No** | **Don't know** |
| 1. Do you think there is no point in trying to get control of your blood sugar well, because the complications of diabetes will occur anyway"? | **Yes** | **No** | **Don't know** |

**Part IV- practice**

| 1. Would you consider treatment if you or one of your family members are found to have diabetes? | **Yes** | **No** | **Don't know** |
| --- | --- | --- | --- |
| 1. Do you do 30-60 mins physical activity daily? E.g. Brisk walking, house activities, climbing staircase. | **Yes** | **No** | **Don't know** |
| 1. Do you check your blood sugar regularly (at least annually)? | **Yes** | **No** | **Don't know** |
| 1. Do you try to avoid refine sugar/sugary foods? | **Yes** | **No** | **Don't know** |

**Thank you!**
